# Clinical Significance of the 5T;12TG Genotype in Pediatric CFSPID: A Retrospective Study

**DOI:** 10.3390/children12060778

**Published:** 2025-06-14

**Authors:** Ana Morales-Tirado, Enrique Blitz-Castro, Saioa Vicente-Santamaría, Carmen Luna-Paredes, Enrique Salcedo-Lobato, Ana Tabares-González, Celia Gascón-Galindo, Simon Boutry, Adelaida Lamas-Ferreiro

**Affiliations:** 1 Cystic Fibrosis Unit, Department of Pediatric Medicine, Ramon y Cajal University Hospital, M-607, Km. 9, 100, Fuencarral-El Pardo, 28034 Madrid, Spain; enrique.blitz@salud.madrid.org (E.B.-C.); saioa.vicente@salud.madrid.org (S.V.-S.); ana.tabares@salud.madrid.org (A.T.-G.); celia.gascon@salud.madrid.org (C.G.-G.); adelaida.lamas@salud.madrid.org (A.L.-F.); 2 Department of Medicine and Medical Specialities, Faculty of Medicine and Health Sciences, University of Alcalá, 28801 Alcalá de Henares, Spain; 3 Instituto Ramón y Cajal de Investigación Sanitaria (Irycis), M-607, Km. 9, 100, Fuencarral-El Pardo, 28034 Madrid, Spain; 4 Cystic Fibrosis Unit, Department of Pediatric Medicine, Doce de Octubre University Hospital, Av. de Córdoba, s/n, Usera, 28041 Madrid, Spain; cluna@salud.madrid.org (C.L.-P.); enrique.salcedo@salud.madrid.org (E.S.-L.); 5 School of Life Sciences, Ecole Polytechnique Fédérale de Lausanne, Station 1, 1015 Lausanne, Switzerland; simon.boutry@uclouvain.be; 6 Swiss Institute of Bioinformatics, Station 1, 1015 Lausanne, Switzerland

**Keywords:** cystic fibrosis inconclusive diagnosis, 5T;12TG, CFTR, neonatal screening, sweat test, children, modulators, ETI

## Abstract

**Background:** One of the most common genetic variants among individuals with cystic fibrosis screen-positive inconclusive diagnosis (CFSPID) is 5T;12TG. Classified as having “varying clinical consequences” (VVCC), it may produce a wide spectrum of CF phenotypes when combined in trans with a pathogenic variant on the other CFTR allele, ranging from asymptomatic cases to CFTR-related disorders (CFTR-RD) or classical cystic fibrosis (CF). The 5T;12TG variant is currently eligible for modulator treatment in the United States. **Methods:** We conducted a retrospective analysis of CFSPID children born between July 2009 and June 2023 in the Community of Madrid (Spain) who carried at least one 5T;12TG variant in trans with another CFTR variant. Data collected included trends in sweat chloride (SC) values, respiratory and digestive symptoms, lung function by spirometry, microbiological findings in nasopharyngeal aspirates, anthropometric data, and fecal elastase levels. **Results:** Twenty-one children (52.3% females; median age: 4.66 years [IQR 3.6–6.9]) were included. Eighteen had 5T;12TG in trans with a CF-causing variant (CFc), two had another VVCC variant, and one had a variant of unknown significance (VUS). After a median follow-up of 3.45 years [IQR 1.4–4.3], all the children remained asymptomatic. However, SC values rose to intermediate levels in nine (42.8%) of the children. No isolates of Pseudomonas aeruginosa were identified. Lung function and pancreatic markers remained normal. **Conclusions:** This is the first Spanish cohort of children with CFSPID and the 5T;12TG allele. Although clinical symptoms did not manifest during childhood, the SC value increased to intermediate values in 42.8% of the cohort, so these may require long-term follow-up to observe conversions to CFTR-RD or CF. The potential initiation of modulator therapy based solely on SC levels or emerging symptoms warrants careful consideration.

## 1. Introduction

Cystic fibrosis (CF) is a chronic, progressive, multi-organ disease due to the presence of pathogenic variants in both alleles of the Cystic Fibrosis Transmembrane Conductance Regulator (CFTR) gene, which encodes a homonymous protein [1]. Its dysfunction results in altered ion transport, leading to dehydration of the liquid surface of the epithelia and dysfunction of the exocrine glands where it is expressed, among which are the respiratory tract, sweat glands, gastrointestinal tract, liver, biliary tract, and the male reproductive system [2].

The newborn bloodspot screening (NBS) program for cystic fibrosis (CFNBS) in Madrid was implemented in 2009. The protocol begins with the measurement of immunoreactive trypsinogen (IRT) levels from the initial dried blood spot. A first IRT cutoff of 44 ng/mL is applied; if this value is exceeded, a second IRT measurement is performed on the same sample. If the average of the two determinations is greater than 50 ng/mL, a CFTR variant panel is conducted [3]. If one or more CFTR variants are identified, the screening is considered positive, and the case is referred for a specialized CFNBS clinical consultation. Conversely, if no variants are detected, a second blood sample is collected for repeat IRT testing. This second test is considered positive if the IRT level exceeds 35 ng/mL. The variants are classified into four types: CF-causing (CFc) variants, variants of varying clinical consequences (VVCCs), variants of uncertain significance (VUSs), or non-CF-causing (nCFc) variants [4]. The third and final step is a sweat chloride (SC) test at a CFNBS Reference Unit. Possible outcomes of this CFNBS include false positives, healthy carriers, CF diagnoses, and children who screen positive but who do not have a conclusive diagnosis, termed “CF screen-positive inconclusive diagnosis” (CFSPID) in Europe or “CFTR-Related Metabolic Syndrome”(CRMS) in the United States [5].

The consensus definition of CSPID is an asymptomatic infant with an abnormal CF-NBS result and (a) SC < 30 mmol/L and two CFTR gene variants, of which at least one is a VVCC or (b) SC ≥ 30–59 mmol/L and one or no CFc variants [5]. This condition could change if their CFTR variants ever reclassify as evidence evolves, or if they ever shift to pathological SC levels or have symptoms (converting to CF if they have multi-organ symptoms or to a CFTR-related disorder (CFTR-RD) if they have isolated symptoms like congenital bilateral absence of the vas deferens (CBAVD)) [6]. Therefore, regular clinical evaluations by CF specialists in accordance with international protocols are essential to detect the onset of symptoms [7,8].

One of the most frequent VVCCs carried by people with CFSPID (pwCFSPID) is 5T;12TG, which has a prevalence of around 10% in the French general population [9]. The 5T variant consists of five contiguous thymidines at the 3′ end of intron 8 of the CFTR gene [10]. Its presence alters exon 9 skipping, and the number of TG repeats correlates with the amount of skipping, resulting in greater or lower functional CFTR protein levels. The 12TG repeat, associated with a higher percentage of altered splicing, gives rise to a great variability of expression among people with the same haplotype, who range from being asymptomatic to having CFTR-RD or CF [11]. The predominant clinical feature is CBAVD in males, but cases with multi-organ symptoms have been described as in CF [12].

In December 2024, the FDA approved the modulator Elexacaftor–Tezacaftor–Ivacaftor (ETI) as a treatment for people with CF carrying the 5T;12TG variant on at least one allele [13]. This opens up the debate about the future treatment that people with CF with this variant may receive, either because they are symptomatic or because they simply have elevated SC levels.

This study aimed to describe the clinical course of a Spanish cohort of individuals with the 5T;12TG CFTR allele combined in trans with alleles of other types: CFc, VVCC, or VUS.

## 2. Materials and Methods

This observational multicenter study included the two CFNBS Centers in the Community of Madrid (Spain): Ramon y Cajal University Hospital and Doce de Octubre University Hospital.

The study population included pwCFSPID born between July 2009 and June 2023 who had at least one 5T;12TG variant in trans with another variant in the CFTR gene. All of them met the definition of CFSPID [5]: they were detected by CFNBS, by an IRT/DNA/2nd IRT strategy, and a posteriori SC test. The CFTR variant panel was the Elucigene CF-EU2v1 kit (Elucigene, UK) together with the Elucigene CF Iberian panel (with 12 additional variants for the Spanish, Portuguese, and Hispanic population) [14].

Clinical data and outcomes for 21 children were updated until June 2024 to evaluate the trend in SC and the clinical course. They all underwent regular clinical follow-ups yearly, according to the existing ECFS standard of care [5]. The Ethical Committee of both centers approved the study, and parents consented to participate in the database of both hospitals.

The CFNBS identified both variants in all the children except one, in whom the panel did not find the L206W variant, so a whole CFTR gene analysis was subsequently conducted. As previously mentioned, we classified the CFTR variants with reference to the CFTR2 and the CFTR-France databases [5,6] as either CFc, VVCC, VUS, or nCFc.

SC values < 30 mmol/L were considered normal, those ≥30–59 were considered intermediate, and those ≥60 mmol/L were positive. A diagnosis of conversion to CF was established if SC values ≥ 60 mmol/L were recorded on two occasions or if the person developed symptoms consistent with CF. It was reclassified to CF if databases revealed new information about their CFTR variants. CFTR-RD was considered if the infant developed a clinical feature consistent with a CFTR-RD but did not meet a CF diagnosis. Pulmonary exacerbations were defined according to CF Foundation criteria [15]. Lung function was measured by the forced expiratory volume in 1 s (FEV_1_) in cooperative children and analyzed by the *z*-score, following ATS guidelines [16,17]. Anthropometric measures (weight, height, and BMI) were also analyzed using *z*-scores. Fecal elastase samples were collected, with <200 μg/g considered the value of pancreatic insufficiency (PI).

Data were checked for normality by the Shapiro–Wilk test (n < 50). Statistical analysis was performed, and based on this, non-parametric tests were conducted. Correlations were assessed with the Spearman rank coefficient, while the Kruskal–Wallis test was used for comparisons between groups, and the Fisher exact test was used for categorical variables. The level of statistical significance was expressed as a *p*-value and considered statistically significant if it was <0.05. If data are represented as medians or means, a measure of deviation is indicated as a range, interquartile range (IQR), or standard deviation (SD).

## 3. Results

### 3.1. Generalities

Complete data were collected from 21 children (52.3% females) carrying the 5T;12TG variant in one allele of the CFTR gene, with a median age of 4.66 years [IQR 3.6–6.9] at the end of the study. Among them, 12 carried the genotype 5T;12TG/F508del (group 1), 6 carried another CFc different from F508del (group 2), 2 had another VVCC in trans with 5T;12TG (group 3), and 1 carried a VUS, 1181+1.6kbA>G (group 4). The main characteristics of each group are presented in Table 1.

The two pathogenic variants were detected by the initial NBS panel in all the children except for one child (L206W/5T;12TG), for whom the L206W variant was not found in the first screening. Therefore, the diagnosis of CFSPID was made from the first month of life in all of them, except for this child, who was diagnosed at five months old after a complete genetic study of him and his parents.

The median IRT was 70.76 ng/mL [IQR 55.9–100.5]. The IRT value was not statistically superior in any group (*p*-value 0.729). The median SC value for the first month was 15.5 mmol/L [IQR 13–22]. There was no correlation between the IRT and the first month’s SC value (*p*-value 0.883). Two children presented intermediate SC values from the first month of life: 30.5 and 38.5 mmol/L. During follow-up, another 7 children had an intermediate SC value at some point, leaving 12 with a consistently negative test (57.14%). Based on our data, the IRT did not significantly affect the SC values (*p*-value 0.4; OR 0.989). The evidence was also not strong enough to conclusively say that the first-month value of SC affects the posterior SC value (*p*-value 0.081; OR 1.15). The number of intermediate values for SC was not statistically superior in any group (*p*-value 0.587).

At the clinical level, there were no symptoms compatible with CF or CFTR-RD, so there were no conversions; all of them maintained the diagnosis of CFSPID.

At the end of the study, 14 continued with the usual annual follow-up in the Unit, while 7 children (33%) discontinued the follow-up during the study period.

### 3.2. Group 1: Genotype 5T;12TG/F508del

Twelve children were identified as having the 5T;12TG/F508del genotype. Of these, only seven continued with the medical check-ups (the rest were lost to follow-up), so we only had data from one child over six years old in the observation period.

Regarding the IRT for this group, the highest value of 214 ng/mL was from a boy who had normal SC levels and who remained asymptomatic.

**Figure 1:**
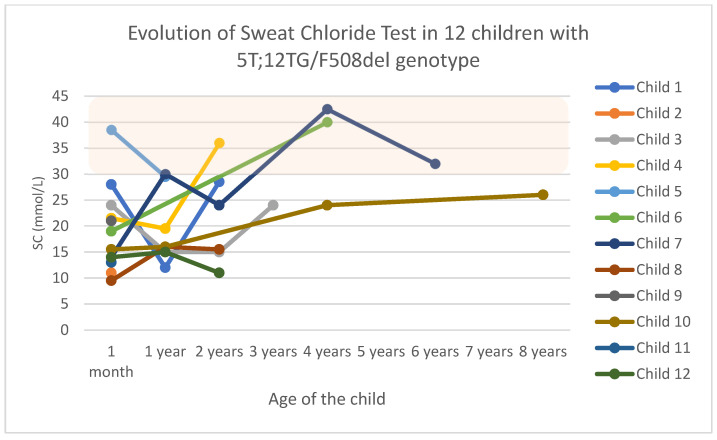
Evolution of sweat chloride test results in 12 children with the F508d/5T;12TG genotype. Abbreviations: SC, sweat chloride.

SC levels remained within normal values for eight children, with four having intermediate values at some point (33.3%): one at the first visit, one at one year old, another at two years old, and the last child at four years old (see Figure 1). The only child with intermediate SC levels from the onset discontinued medical appointments at one year of age.

Clinically, one boy with an IRT of 71.5 ng/mL and consistently normal SC levels had right lower lobe, middle lobe, and left lower lobe lateral segment pneumonia with right costophrenic sinus impingement (minimal pleural effusion) at 3 years of age. He required hospitalization and was treated with oxygen therapy, antibiotherapy, and bronchodilators, with a favorable evolution. Previously, he had recurrent wheezing that was treated with inhaled budesonide and montelukast, which had been withdrawn due to a good evolution. He remained asymptomatic after the pneumonia. He had no digestive symptoms and had normal anthropometric values. No nasopharyngeal aspirate cultures (NFCs) or elastase were recorded in his clinical records.

The only available lung function was from a girl with an IRT of 48 ng/mL and intermediate SC values (child 7 in Figure 1). There were no respiratory or digestive symptoms reflected in her clinical records, and isolated growths of *Streptococcus pneumoniae* (*S. pneumoniae*), *Haemophilus influenzae* (*H. influenzae*), and *Moraxella catarrhalis* (*M. catharralis*) were found in her NFCs. She underwent a spirometry test as well as the other complementary tests (fecal elastase, abdominal ultrasound, liver elastography), which were all within normal values.

The two individuals with out-of-range weight *z*-scores were a child with −3 SD at the first visit with no history of prematurity and who did not return for follow-up (child 2 in Figure 1), and another with +2.20 SD, who was also lost to follow-up at one year of age (child 5 in Figure 1).

### 3.3. Group 2: Genotype 5T;12TG/Cystic Fibrosis-Causing Variant (Other than F508del)

There were six cases in which the genotype included the 5T;12TG variant with a CFc other than F508del: G542X, L206W, 1811+1634A->G (n = 2 siblings), 3120+1G->A, and V232D (see characteristics in Table 1).

The child with the highest IRT was the G542X carrier with 124 ng/mL, who was asymptomatic and had normal SC levels.

**Figure 2:**
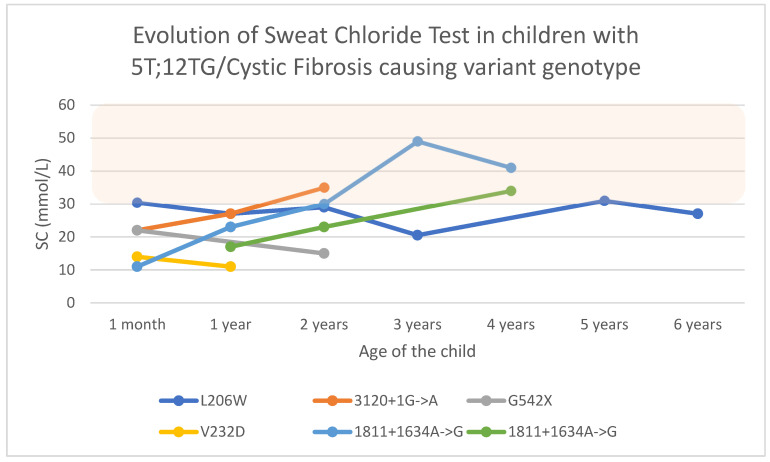
Evolution of sweat chloride test results in children with F508del and a cystic fibrosis-causing variant other than F508del. Abbreviations: SC, sweat chloride.

Four of the six children in the group had intermediate SC levels on some occasions (66.6%), including one child (L206W) who already showed this level at the first visit and who maintained values of around 30 mmol/L over time (see Figure 2). The siblings with 1811+1634A->G and the one with 3120+1G->A are the other three children.

The child with 5T;12TG/L206W was diagnosed with asthma after a history of coughing with exercise since he was three years old and an unusual frequent expectoration. His lung function was within reference values. He had been treated with bronchodilators and inhaled corticosteroids at medium doses. This child had chronic bronchial colonization by *H. influenzae* and *methicillin-sensitive Staphylococcus aureus* (MSSA), and intermittent colonization by *M. catharralis*. In addition, he presented with primary colonization by *Escherichia coli* at 7 months of age and *Stenotrophomonas maltophilia* (*S. maltophilia*) at 13 months of age. Both resolved spontaneously without treatment. His IRT was 95 ng/mL, with intermediate SC levels. Digestive symptoms were absent, with normal complementary tests (elastase, abdominal ultrasound, and hepatic elastography).

In this group, two of the children had out-of-ordinary weight *z*-score values: one above with +2.10 SD and one below with −2.5 SD. The latter child was treated with ranitidine for gastroesophageal reflux. Both had normal elastase levels.

### 3.4. Group 3: Genotype 5T;12TG/Variant of Varying Clinical Consequences

Two girls had double-variant VVCCs, which consisted of one R117H and one homozygous 5T;12TG. The latter child presented a very high IRT of 120 ng/mL at diagnosis. They were the oldest age group, being 7 and 13 years, respectively, at the end of the observation period. Both had normal SC levels and remained asymptomatic (see Table 1). Only the R117H carrier underwent spirometry, which was within normal values.

### 3.5. Group 4: 5T;12TG/Variant of Unknown Clinical Significance at Present

One girl had a variant not described in the U.S. and French genetic databases [4,18], 1181+1.6kbA>G, but the Elucigene CF Iberian NBS screening panel detected it. The girl had an IRT of 55.6 ng/mL and a normal SC value (14 mmol/L) at the first visit. Her SC value did not exceed 30 mmol/L until nearly four years of age (32.5 mmol/L), and she maintained the same value at the next annual check-up. Clinically, she had a pulmonary exacerbation (PEx) at 2.5 years of age that required a visit to the emergency department, and prophylactic antibiotic therapy was prescribed until the microbiological result was known, which was finally positive for the *Influenza* virus. NFCs showed intermittent bronchial colonization by *S. pneumoniae*, and she once had a primary colonization with *S. maltophilia*, which did not require treatment. She maintained weight and height values above +2 SD. Out of five elastase determinations, one was in the PI range, which was considered a laboratory error because subsequent results confirmed normal pancreatic function.

## 4. Discussion

To enhance our understanding of the pathogenic potential of the 5T;12TG allele, this study presents the clinical course of 21 children diagnosed with CFSPID carrying this variant. We grouped the genotypes into four categories based on two main considerations: First, we followed the established classification of CFTR variants (CFc, VVCC, and VUS), as per official guidelines [4]. Second, within the CFc group, we identified a predominance of the F508del variant, which is not only the most frequent CFc in our cohort but also one that has been the focus of specific studies [11]. For these reasons, we considered it appropriate to separate F508del carriers into a distinct subgroup to allow for more meaningful interpretation and comparison. While some interindividual variability may exist within these categories, we believe this classification balances clinical relevance with analytical clarity. Due to the small number of individuals in certain subgroups, particularly groups 3 and 4, the calculated statistical power was considerably below the conventional threshold of 0.8, reinforcing the need for a cautious interpretation of the subgroup comparisons.

No significant differences in IRT levels were observed across the genotypic groups, nor was a correlation established between IRT and either initial or evolving SC values. Therefore, in our cohort, the IRT is not a predictive marker. These findings contrast with those of Padoan et al., who reported that seven infants with the 5T;12TG allele exhibited elevated IRT levels, all of whom had an intermediate SC value in the first months of life [9].

It is well documented that an initial intermediate SC value increases the risk of conversion to CF over time [19,20]. In our cohort, the *p*-value of 0.08 suggests a weak trend, indicating that the SC value in the first month might influence later SC outcomes.

Regarding SC evolution, the CFTR2 database includes 608 individuals with a median SC of 57 mmol/L, which is within the intermediate range [4]. A large cohort study by Tosco et al. of 129 individuals with the F508del/5T;12TG genotype found that 29 were diagnosed with CF directly based on their SC values > 60 mmol/L [11]. An additional 10 conversions occurred among the CFTR-RD and CFSPID people due to persistent positive SC values after a median follow-up of 6.7 years. Therefore, 39 out of the 129 (30%) individuals were converted based on the SC criteria, highlighting the potential for progression. In our study, none of the children reached the CF diagnostic SC, but 42.8% developed intermediate levels, suggesting a need for extended surveillance to assess possible conversions.

Similar to our group 3 with the 5T;12TG/VVCC genotype, Tosco et al. reported on 27 individuals (1 CF, 8 CFTR-RD, and 18 CFSPID), with one diagnosed as CF at the outset due to a positive SC test and bronchiectasis, and one CFSPID converting to CF (5.5%) due to a pathological SC test result (112 mEq/L) during the four years of follow-up [21]. Their percentage of individuals with intermediate SC was 35.3% among CFSPIDs and 62.5% among CFTR-RFs. The two cases of our cohort retained normal SC values and their CFSPID status throughout the follow-up period.

The child with the VUS 1181+1.6kbA>G exhibited rising SC values into the intermediate range. Previous reports associate this variant with severe CF phenotypes and high SC values of around 98 mmol/L, underscoring the importance of continued monitoring [22].

Clinically, our findings align with the existing international literature suggesting that most individuals with this genotype remain asymptomatic during childhood [8]. Although CBAVD is the most common phenotype, some individuals exhibit multisystem involvement [12]. For example, Noone et al. described a F508del/5T;12TG woman with recurrent pneumonia since childhood and SC values that were consistently > 70 mmol/L [23]. Similarly, Praticó et al. reported on a 15-year-old boy with hypertransaminasemia, hepatomegaly, and jaundice, who was later diagnosed with CF based on F508del/5T;12TG and a positive SC test [24]. Dray et al. described a 22-year-old woman with F508del/5T;2TG who had recurrent bronchitis, nasal polyps, and pancreatitis and who had pathological SC levels [25]. Tosco et al. examined a cohort of 129 people with F508del/5T;12TG and showed that 30 were diagnosed with CF with symptoms such as CBAVD (66.7%), bronchiectasis (36.6%), PI (10%), and pancreatitis (3.3%). Additionally, 22% of CFTR-RD individuals converted to CF due to multi-organ symptoms [11]. In our study, the pulmonary symptoms, when present, were generally mild and self-limited, and all the children retained normal pancreatic function, consistent with that reported by other authors [26].

Several cases have also been described in homozygous 5T;12TG people. Noone et al. presented a woman with frequent respiratory infections since adolescence, sinusitis, diffuse bronchiectasis who had positive cultures for *Pseudomonas aeruginosa* (*Pa*) and intermediate SC values [23]. Another case involved a 17-year-old boy with recurrent pancreatitis since the age of 13 years, who had a positive SC of >80 mmol/L [27]. A third case described a 3-year-old boy with recurrent bronchitis and pneumonia and poor weight gain, despite negative NBS and SC values [28]. Tosco et al. studied a cohort of 27 individuals with 5T12TG/VVCC and reported eight diagnoses of CFTR-RD based on their CF family history (50%), respiratory symptoms (25%), recurrent pancreatitis (12.5%), and CBAVD (12.5%), and one was diagnosed with CF from the outset due to bronchiectasis and CBAVD with a positive SC result; this corresponds to 33.3% symptomatic people in the cohort) [20]. In our study, the two female children showed no clinical alarm parameters, despite being the oldest at the end of the follow-up (mean age: 9.9 years). Symptoms may still appear in adulthood, although this is less likely in females given the absence of CBAVD.

Finally, we included one child with the 1181+1.6kbA>G variant, which has not been described in the U.S. and French databases [4,18]. A 1995 Spanish article described this variant in 21 Spanish and one German chromosome, associating it with a severe CF phenotype indistinguishable from those individuals homozygous for F508del [22]. Our case underscores the importance of ongoing monitoring given the rising SC values and potential symptom development.

No cases of *Pa* or mycobacterial colonization were observed in our cohort. As noted by Terlizzi et al. in one of their recent publications, clinical practice varies greatly between centers [29], so any colonization was treated in our population.

Limitations of our study include its retrospective design, the small overall sample size in some subgroups, and loss to follow-up, mainly due to the COVID-19 pandemic, which significantly disrupted routine clinical monitoring. These factors restrict the ability to draw definitive conclusions, particularly for the less-represented genotypes. Additionally, although our CFNBS program began in 2009, most CFSPID diagnoses occurred over the past decade, following updates to the international consensus guidelines [5]. Consequently, longitudinal clinical data remain limited for certain specific genetic combinations. Nevertheless, our cohort provides a representative snapshot of the current genetic diversity and management approaches for CFSPID children in the Madrid region. Importantly, this study has led to efforts to re-establish contact with children lost to follow-up, facilitating future data collection and longer-term studies.

Our data do not suggest a higher risk based on the pathogenic category of the other allele. This leads to the fact that 5T;12TG itself has recently been included as one of the non-F508del variants receiving FDA approval as an ETI-responsive variant [13]. In the absence of international consensus regarding the management of asymptomatic children carrying the 5T;12TG variant, this study underscores the need for a cautious and individualized approach. The most common reason for treatment would be asymptomatic children converting to CF due to a positive SC test; however, a variability of approximately 20% in SC values between different samples from the same person has been documented [30,31]. Furthermore, there are still few long-term cohorts, and most children do not develop symptoms during their early life, raising the question of whether to treat asymptomatic children who carry this variant [32]. This issue involves significant ethical and economic concerns, as initiating treatment in asymptomatic individuals at a very early stage may lead to over-medicalization and lifelong healthcare costs. These people typically do not experience severe PEx, while their spirometric and nutritional parameters are generally within normal ranges, and their NFCs often show a usual microbiota composition. Therefore, it is important to define which clinical parameters, beyond SC values, should be monitored during follow-up. One option is to incorporate additional pulmonary function tests with reliable results at younger ages, such as Lung Clearance Index testing, or low-radiation imaging techniques to detect early lung damage. In such cases, objective findings could support more informed treatment decisions. On the other hand, the CF scientific community has long supported early intervention and prevention, particularly in pediatric populations, where irreversible organ damage might be preventable. Consequently, further research is warranted to better define the risk–benefit profile of early ETI initiation in this group, especially for the assumption that CFSPID infants who convert to CF based on elevated SC values may eventually develop organ involvement. Another ongoing debate is whether treatment should be indicated in CFTR-RD, as the most frequent clinical manifestation is isolated CBAVD. Our findings emphasize the importance of long-term follow-up and suggest that decisions regarding modulator therapy should not rely exclusively on biochemical changes. Instead, a more comprehensive evaluation incorporating clinical, functional, and imaging parameters is warranted to ensure that treatment initiation is both justified and beneficial.

## 5. Conclusions

This study provides valuable insights into the short-term natural history of CFSPID in children carrying the 5T;12TG variant. Notably, this is the first published Spanish cohort of CFSPID children, and it contributes novel data showing the significant changes in SC values over just a few years of follow-up, with a 42.8% of the children reaching intermediate levels. No conversions to CF or CFTR-RD were observed, which reinforces the existing literature by confirming the absence of early clinical manifestations during childhood. However, the variability in SC values highlights the potential for future disease progression and underscores the need for long-term surveillance to detect late-onset symptoms. The decision to initiate modulator therapy should be carefully considered and based on both biochemical and clinical evidence. Inclusion of these individuals in national and international registries is crucial to enhancing our understanding of their clinical trajectory and to guide future management strategies.

## Figures and Tables

**Table 1:** Clinical characteristics of CFSPID children carrying a 5T;12TG allele.

Genotypes (n)	5T;12TG/F508del (n = 12)	5T;12TG/CFc non-F508del (n = 6)	5T;12TG/VVCC (n = 2)	5T;12TG/VUS (n = 1)
Allele non-5T;12TG (n)	F508del (n = 12)	1811+1634A->G (n = 2) L206W (n = 1) G542X (n = 1) 3120+1G->A (n = 1) V232D (n = 1)	5T;12TG (n = 1) R117H;7T (n = 1)	1181+1.6kbA>G (n = 1)
Female (%)	5 (41.7%)	3 (50%)	2 (100%)	1 (100%)
Preterm birth (%)	1 (8.3%)	2 (33.3%)	0 (0%)	0 (0%)
IRT median [IQR] (ng/mL)	71.13 [54.8–78.7]	80.55 [61.8–110.5]	89.5 [59–120]	55.6
CFNBS diagnosis by the initial panel (%)	12 (100%)	5 (83.3%)	2 (100%)	1 (100%)
Age at first visit, median [IQR] (days)	27 [21–41]	44.5 [35.5–65.2]	38 (n = 1)	21
Age at the end of the study, median [IQR] (years)	4.25 [3.4–5.3]	4.59 [4.3–7.1]	9.9 [7–12.9]	4.76
Conversion to CF (%)	0 (0%)	0 (0%)	0 (0%)	0 (0%)
Conversion to CFTR-RD	0 (0%)	0 (0%)	0 (0%)	0 (0%)
First visit SC, median [IQR] (mmol/L)	17.2 [13.2–23.4]	22 [16–28.3]	11.5 [11–12]	14
Number of children with intermediate SC at the first visit (%)	1 (8.33%)	1 (16.7%)	0 (0%)	0 (0%)
Number of children with intermediate SC at some point during follow-up (%)	4 (33.3%)	4 (66.6%)	0 (0%)	1 (100%)
Number of children with a weight *z*-score in the range +/− 2SD (%)	10 (83.3%)	4 (66.7%)	2 (100%)	0 (0%)
Number of children with a height *z*-score in the range +/− 2SD (%)	12 (100%)	6 (100%)	2 (100%)	0 (0%)
Last FEV_1_ *z*-score, median (n)	−0.4 (n = 1)	0.12 (n = 1)	0.19 (n = 1)	-
Respiratory symptoms (%)	Wheezing (16.7%) Bilateral pneumonia (8.3%)	Bronchiolitis (33.3%) Asthma (16.7%)	0 (0%)	PEx (50%)
Hospitalizations (%)	2 (16.7%)	0 (0%)	0 (0%)	0 (0%)
Bronchial colonization isolations (%)	*H. influenzae* (50%) *M. catarrhalis* (50%) MSSA (33.3%) *S. pneumoniae* (25%) *A. baumanii* (8.3%)	MSSA (33.3%) *H. influenzae* (16.7%) *M. catarrhalis* (16.7%) *S. pneumoniae* (16.7%) *S. maltophilia* (16.7%) *Escherichia Coli* (16.7%)		MSSA (50%) *S. pneumoniae* (50%) *S. maltophilia* (50%)
Digestive symptoms (%)	Constipation (8.3%)	GE reflux (16.7%) Constipation (16.7%)	0 (0%)	0 (0%)
Pancreatic insufficiency (%)	0 (0%)	0 (0%)	0 (0%)	0 (0%)
Physiotherapy Inhaled corticosteroid	0 (0%) 2 (16.7%)	0 (0%) 1 (16.7%)	0 (0%) 0 (0%)	0 (0%) 0 (0%)

Abbreviations: CFc, cystic fibrosis-causing variant; VVCC, variant of varying clinical consequences; VUS, variant of unknown significance; IRT, immunoreactive trypsinogen; CFNBS, cystic fibrosis newborn screening; SC, sweat chloride; FEV_1_, forced expiratory volume in 1 s; PEx, pulmonary exacerbation; *H. influenzae*, *Haemophilus influenzae*; *M. catarrhalis*, *Moraxella catarrhalis*; MSSA, *methicillin*-*sensitive Staphylococcus aureus*; *S. pneumoniae*, *Streptococcus pneumoniae*; *A. baumanii*, *Acinetobacter baumanii*; *S. maltophilia*, *Stenotrophomonas maltophilia*; GE, gastroesophageal reflux.

## Data Availability

The data supporting the findings of this study are available from the corresponding author upon reasonable request, as indicated in the informed consent..

## Abbreviations

The following abbreviations are used in this manuscript:

CF	Cystic fibrosis
CFTR	Cystic Fibrosis Transmembrane Conductance Regulator
CFSPID	CF screen-positive inconclusive diagnosis
SC	Sweat chloride
CFTR-RD	CFTR-related disorder
NBS	Newborn bloodspot screening
IRT	Immunoreactive trypsinogen
CFc	CF-causing variants
VVCC	Variants of varying clinical consequences
VUS	Variants of uncertain significance
nCFc	Non-CF-causing variant
FEV1	Forced expiratory volume in 1 s
PI	Pancreatic insufficiency
IQR	Interquartile range
ETI	Elexacaftor–Tezacaftor–Ivacaftor modulator treatment
